# Laccase Production Optimization from Recombinant *E. coli* BL21 Codon Plus Containing Novel Laccase Gene from *Bacillus megaterium* for Removal of Wastewater Textile Dye

**DOI:** 10.3390/molecules29235514

**Published:** 2024-11-22

**Authors:** Zannara Mustafa, Ikram ul Haq, Ali Nawaz, Abdulrahman H. Alessa, Muhammad Nauman Aftab, Ahmad A. Alsaigh, Aziz ur Rehman

**Affiliations:** 1 Dr. Ikram ul Haq Institute of Industrial Biotechnology, GC University, Lahore 54600, Pakistan; zannaramustafa@gmail.com (Z.M.); ali.nawaz@gcu.edu.pk (A.N.); nauman535@yahoo.com (M.N.A.); iamaziz435@gmail.com (A.u.R.); 2 School of Applied Science, University of Huddersfield, Huddersfield HD1 3DH, UK; 3 Department of Biology, Faculty of Science, University of Tabuk, Tabuk 47512, Saudi Arabia; alessiabdulrahman@gmail.com; 4 Department of Biology, Faculty of Science, Umm Al-Qura University, Makkah 24382, Saudi Arabia; aassaigh@uqu.edu.sa

**Keywords:** recombinant laccase, textile, dye, optimization, effluent, precipitation, *Bacillus megaterium*

## Abstract

The aim of the present research was the efficient degradation of industrial textile wastewater dyes using a very active cloned laccase enzyme. For this purpose, potent laccase-producing bacteria were isolated from soil samples collected from wastewater-replenished textile sites in Punjab, Pakistan. The laccase gene from locally isolated strain LI-81, identified as *Bacillus megaterium*, was cloned into vector pET21a, which was further transformed into *E. coli* BL21 codon plus. The optimized conditions for the increased production of laccase include fermentation in a 2% glucose, 5% yeast extract and 250 mg/L CuSO_4_ medium with pH 7.5; inoculation with 5% inoculum; induction with 0.1 mM IPTG at 0.5 O.D.; and incubation for 36 h at 37 °C. The crude enzyme produced was employed for the removal of commercially used textile dyes. The dyes were quickly precipitated under optimized reaction conditions. Rose bengal, brilliant green, brilliant blue G, Coomassie brilliant blue R and methylene blue were precipitated at rates of 10.69, 54.47, 84.04, 78.99 and 7.40%, respectively. The FTIR and UV–Vis spectroscopic analyses of dyes before and after confirmed the chemical changes brought about by the cloned laccase that led to the dye removal.

## 1. Introduction

The use of textile dyes results in the discharge of toxic and carcinogenic compounds into water bodies. It is estimated that globally, over 10,000 types of textile dyes are used, with the selection of dye varying according to the type and nature of the fabric employed. Approximately 0.7 million tons of synthetic dyes are used worldwide each year [1]. During the process of dyeing, the unfixed dyes are washed away as an effluent, which increases their concentration in nearby water bodies [2]. Pakistan, being the eighth-largest exporter of textile products in Asia, requires a cost-effective and environmentally friendly method of treating textile wastewater [3].

The Scopus database has shown an overwhelming increase in interest in the treatment of textile wastewater with the help of laccase (1.10.3.2). Among others, the United States, India, South Korea, China and Mexico are actively involved in the research and application of laccase for textile wastewater treatment [4]. Researchers are introducing genetic modifications to intensify laccase activity [5]. Various manufacturers, like Amano enzyme Co., Ltd., Elgin, IL, USA; Novozymes, Bagsværd, Denmark; Season Chemicals, Taizhou, China; AB Enzymes, Darmstadt, Germany; Genecor Inc., Rochester, NY, USA; and Zytex Pvt. Ltd., Mumbai, India, are producing laccase on a commercial scale [6,7,8].

The treatment of textile wastewater with other physical and chemical methods is not efficient as it is not able to degrade all the dyes [9]. These methods sometimes convert the dyes into more harmful products [10]. In contrast, the oxidation of dyes with laccase is more promising as it acts less specifically on the aromatic ring and has a wide range of substrates. In this way, it has a high potential for the degradation of a large number of textile dyes [11]. This method can also degrade other pollutants like microplastics, pharmaceuticals and endocrine disruptors [12].

Laccase, a dioxygen oxidoreductase, is also called multicopper oxidase. It carries out the oxidation of substrates and yields water by reducing molecular oxygen. Many dimeric, oligomeric, polymeric and cross-coupling products are typically yielded by the reaction [13]. Laccase can catalyze phenolic compounds like Catechol, Resorcinol and Vanillin [14]. It also requires mediators like ABTS to catalyze inaccessible, non-phenolic compounds [15]. The diverse substrate oxidation ability of laccase is due to the copper atoms present in its catalytic region. It has four copper atoms: T1 (type 1), a blue paramagnetic copper atom; T2 (type 2), a non-blue paramagnetic copper atom; and two T3 (type 3) atoms, which are diamagnetic, spin-coupled and paired [16]. Laccase has the ability to withstand harsh pH and temperature conditions. This is due to the carbohydrate portion, which has the responsibility of providing conformational stability and resistance to proteolysis [17].

Laccase has low substrate specificity; therefore, it can act on a variety of substrates [18]. It carries out electron reduction of oxygen into water via the uptake of electrons from the substrate that causes the oxidation of the phenolic substrate [19]. The redox potential of laccase is lower than that of non-phenolic compounds, so it cannot mediate the process of oxidation alone. Thus, low-molecular-weight compounds like 2,2-azino-bis 3-ethylbenzothiaoline-6-sufonic acid (ABTS) and 1-hydroxybenzotriazole (HBT) can act as mediators to facilitate oxidation via laccase [20].

Rose bengal dye is extensively used in the textile, plastic and printing industries [21]. It has very harmful effects, which are worsened by its high solubility in water. It is therefore highly toxic for living beings [22]. Rose bengal dye is also toxic to the human corneal epithelium and causes itchiness, irritation, reddening and blistering [23]. Brilliant green dye is actively used in the wood, silk, rubber and plastic industries. It is also hazardous if it comes into contact with the human body, as prolonged and repeated exposure has the potential to cause targeted organ damage [24]. Sulfur-containing brilliant blue G is extensively used in different industries, including the textile sector, and shows reasonable stability in light, heat and an acidic environment but has a low oxidative stability, making it a good target for laccase [25]. Coomassie brilliant blue R is a triphenylmethane dye [26] that was originally developed to dye wool. Now, it is used in cases where lightfastness is not important, like hectographs, textile applications, printing inks and copying papers [27]. Methylene blue, a commonly used cationic dye for paper and leather, is very persistent, carcinogenic and mutagenic [28].

The present study was conducted to optimize the production of laccase from recombinant *E. coli* BL21 codon plus with the laccase gene from *Bacillus megaterium* and its ability to treat textile dyes. The details of the isolation and cloning of the laccase gene are provided in a previously published research paper [29]. To date, no research paper has reported the cloning and expression of laccase from *Bacillus megaterium*, so the present work can be helpful for other researchers in the future. In the present study, the optimization of various parameters was carried out, which significantly increased the yield.

## 2. Results

### 2.1. Isolation of Laccase-Producing Bacteria from Soil

**Figure 1:**
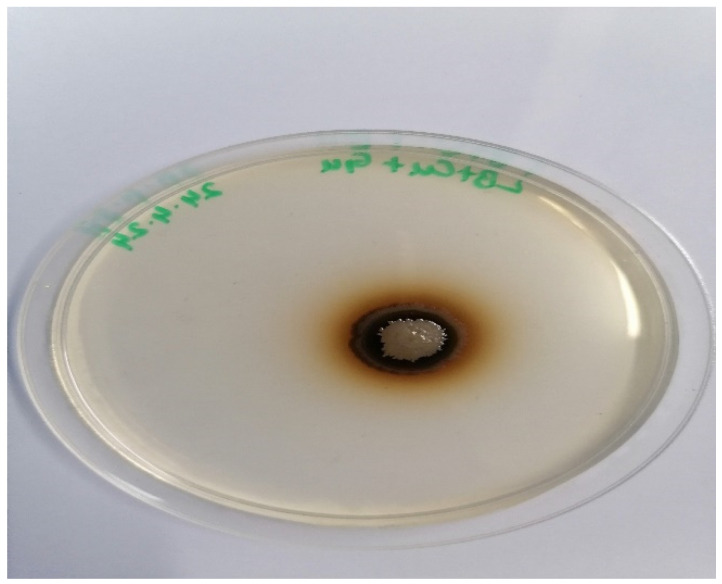
Brown zone produced by laccase-producing bacteria isolated from soil.

Among various isolates obtained from the soil replenished with textile wastewater, the one with the largest zone was selected for the cloning of the laccase gene. The isolate is shown in Figure 1. The 16S rRNA sequencing results showed that the bacterial isolate was *Bacillus megaterium* (accession number: PQ044642).

### 2.2. Cloning of Laccase Gene from Locally Isolated Bacillus megaterium

**Figure 2:**
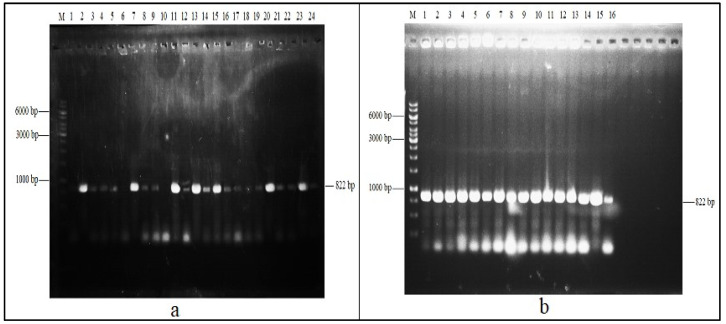
(**a**) Colony PCR of laccase gene in vector PCR 2.1 transformed into DH5α. (**b**) Colony PCR of laccase gene cloned into pET21a further transformed into BL21 codon plus.

The isolated and amplified laccase gene of *B. megaterium* was cloned into PCR vector 2.1 and transformed into DH5α, which was confirmed by the colony PCR, as shown in Figure 2a. This gene was further sub-cloned into pET21a and transformed into *E. coli* BL21 codon plus. The cloning of the laccase gene was confirmed by the colony PCR, and the results are depicted in Figure 2b.

### 2.3. Expression of Recombinant Laccase (rILac)

**Figure 3:**
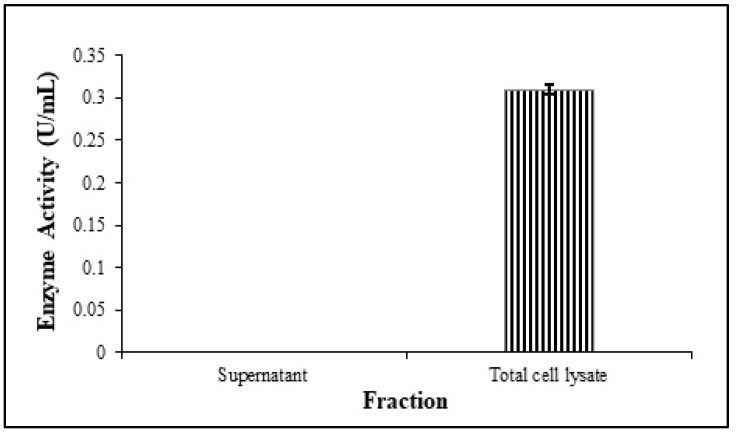
Comparison of rILac activity in different fractions of LB broth culture medium.

It was observed that the rILac was produced intracellularly as the cell lysate showed activity against guaiacol, but the supernatant showed negligible activity (Figure 3). The total cell lysate showed 0.31 U/mL enzyme activity.

### 2.4. Optimization of rILac Production

The following parameters were optimized to increase the production of rILac.

#### 2.4.1. Effect of Medium, Fermentation Time, pH, Carbon Source and Its Concentration

**Figure 4:**
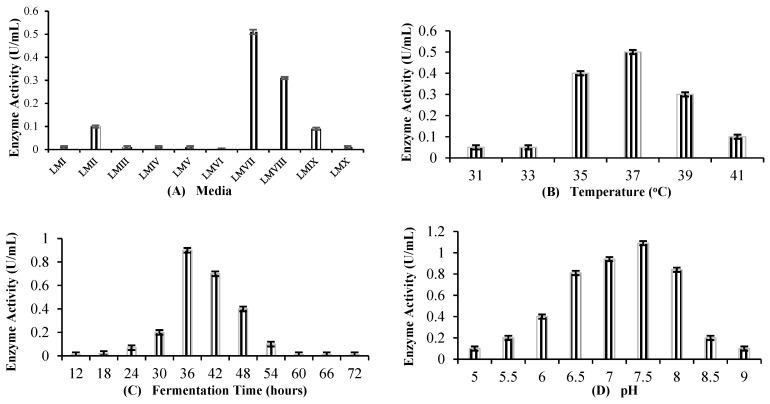

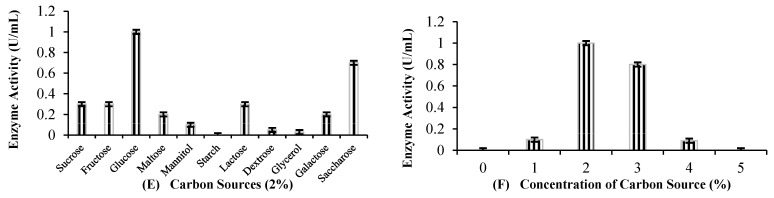
Effect of (**A**): **medium composition** (37 °C, 24 h of fermentation, 7 pH, 2% inoculum, 0.1 mM IPTG, 0.4 O.D., 100 mg/L CuSO_4_); (**B**): **temperature** (YPD-Cu, 24 h of fermentation, 7 pH, 2% inoculum, 0.1 mM IPTG, 0.4 O.D., 100 mg/L CuSO_4_); (**C**): **fermentation time** (37 °C, YPD-Cu, 7 pH, 2% inoculum, 0.1 mM IPTG, 0.4 O.D., 100 mg/L CuSO_4_); (**D**): **pH** (37 °C, YPD-Cu, 24 h of fermentation, 2% inoculum, 0.1 mM IPTG, 0.4 O.D., 100 mg/L CuSO_4_); (**E**): **carbon source** (37 °C, 24 h of fermentation, 0.2% yeast extract, 0.5% peptone, 2% inoculum, 0.1 mM IPTG, 0.4 O.D., 100 mg/L CuSO_4_); (**F**): **concentration of carbon source** (37 °C, YPD-Cu, 24 h of fermentation, 0.2% yeast extract, 0.5% peptone, 2% inoculum, 0.1 mM IPTG, 0.5 O.D., 100 mg/L CuSO_4_).

Among the 10 various types of media used, it was found that LMVII, named YPD-Cu, supported the maximum production of rILac (Figure 4A). The rILac resulted in 0.51 U/mL laccase activity in this medium. The 37 °C temperature was observed to be the optimum temperature at which rILac showed activity of 0.51 U/mL (Figure 4B). The results showed 36 h of fermentation time to be the best for maximum production (Figure 4C). Here, rILac showed 0.9 U/mL enzyme activity. pH 7.5 was the best observed pH for the maximum production of rILac, showing 1.0 U/mL enzyme activity (Figure 4D).

Many types of carbon sources were tested. It was found that glucose, which has already been used, was the best carbon source for the optimum production of rILac. The rIBL21 codon plus resulted in 1.0 U/mL activity, which was the maximum production among the used carbon sources. The results are depicted in Figure 4C. The varying concentrations of glucose were also analyzed to determine the optimum production of rILac. It was found that 2% glucose, which has already been used at the same concentration, resulted in the maximum production of the enzyme (Figure 4D).

#### 2.4.2. Effect of Nitrogen Source, Its Concentration and Inoculum Size

**Figure 5:**
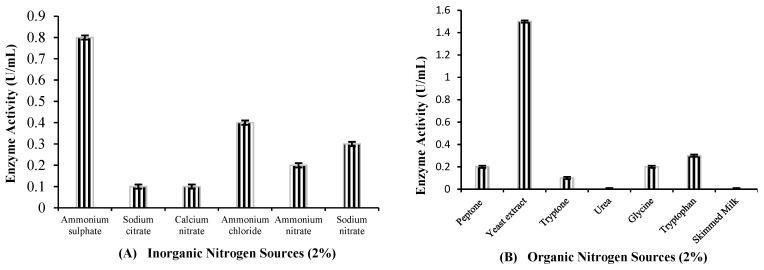

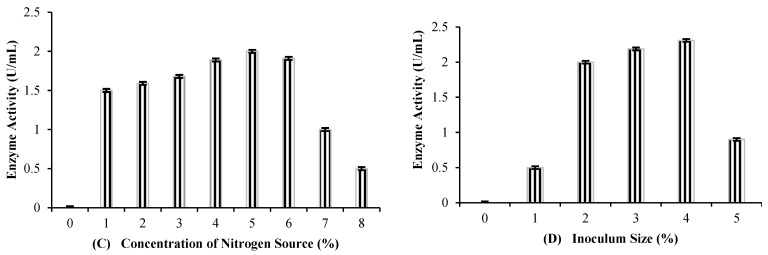
Effect of (**A**): **inorganic nitrogen source** (37 °C, 24 h of fermentation, 2% glucose, 2% inoculum, 0.1 mM IPTG, 0.4 O.D., 100 mg/L CuSO_4_); (**B**): **organic nitrogen source:** (37 °C, 24 h of fermentation, 2% glucose, 2% inoculum, 0.1 mM IPTG, 0.4 O.D., 100 mg/L CuSO_4_); (**C**): **concentration of nitrogen source** (37 °C, 24 h of fermentation, 2% glucose, 2% inoculum, 0.1 mM IPTG, 0.4 O.D., 100 mg/L CuSO_4_); (**D**): **inoculum size:** (37 °C, 24 h of fermentation, 2% glucose, 5% yeast extract, 0.1 mM IPTG, 0.4 O.D., 100 mg/L CuSO_4_).

The inorganic and organic nitrogen sources were separately assessed for their maximum recombinant yield. Among the inorganic nitrogen sources, ammonium sulfate was found to be the best, as shown in Figure 5A, while yeast extract was observed to be the best nitrogen source among the organic nitrogen sources, as depicted in Figure 5B. It was concluded that yeast extract had the maximum recombinant laccase production (1.5 U/mL) among all media by comparing the enzyme production of media with yeast extract and that with ammonium sulfate. The results showed that recombinant laccase production was dramatically increased by adding 5% yeast extract to the fermentation medium. In this case, 2.0 U/mL activity was observed, which declined upon a further increase in the yeast extract concentration (Figure 5C).

An increase in recombinant laccase production was observed by increasing the inoculum size up to 4% (2.31 U/mL). However, the further increase decreased laccase production (Figure 5D).

#### 2.4.3. Inducer Type, Its Concentration, Induction Time and Copper Ion Concentration

**Figure 6:**
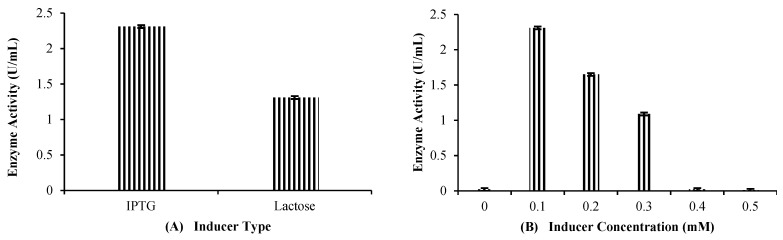

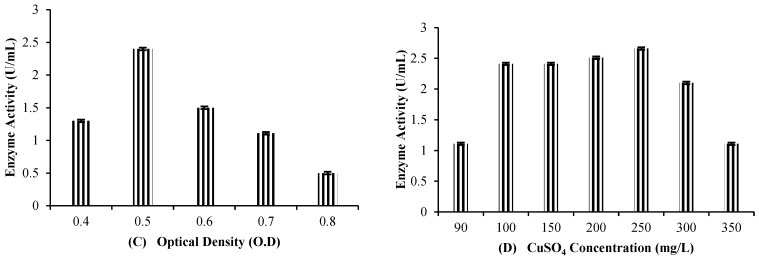
Effect of (**A**): **inducer type** (37 °C, 24 h of fermentation, 2% glucose, 5% yeast extract, 4% inoculum, 0.4 O.D., 100 mg/L CuSO_4_); (**B**): **inducer concentration** (37 °C, 24 h of fermentation, 2% glucose, 5% yeast extract, 4% inoculum, 0.4 O.D., 100 mg/L CuSO_4_); (**C**): **optical density** (37 °C, 24 h of fermentation, 2% glucose, 5% yeast extract, 4% inoculum, 0.1 mM IPTG, 100 mg/L CuSO_4_); (**D**): **CuSO_4_ concentration** (37 °C, 24 h of fermentation, 2% glucose, 5% yeast extract, 4% inoculum, 0.1 mM IPTG, 0.4 O.D.).

By comparing the induction capacity of two inducers, lactose and IPTG, it was found that IPTG was the most suitable inducer (Figure 6A). However, the enzyme activity remained the same (2.31 U/mL) for both inducers. Various concentrations of IPTG were employed to ascertain the best concentration for producing the maximum laccase. The 0.1 mM IPTG concentration was the most effective for the efficient production of laccase (Figure 6B). The same concentration was used in the initial optimization parameters, so the enzyme activity (2.31 U/mL) remained the same.

The most suitable induction time was determined by carrying out induction at different O.D. levels. It was found that 0.5 O.D. was the best for maximum rILac production. Inducing the culture at 0.5 O.D. caused 2.31 U/mL enzyme activity, as shown in Figure 6C. The most effective concentration of copper sulfate for the maximum rILac production was 250 mg/L, at which 2.67 U/mL recombinant laccase was produced, as shown in Figure 6D.

### 2.5. Dye Removal

**Figure 7:**
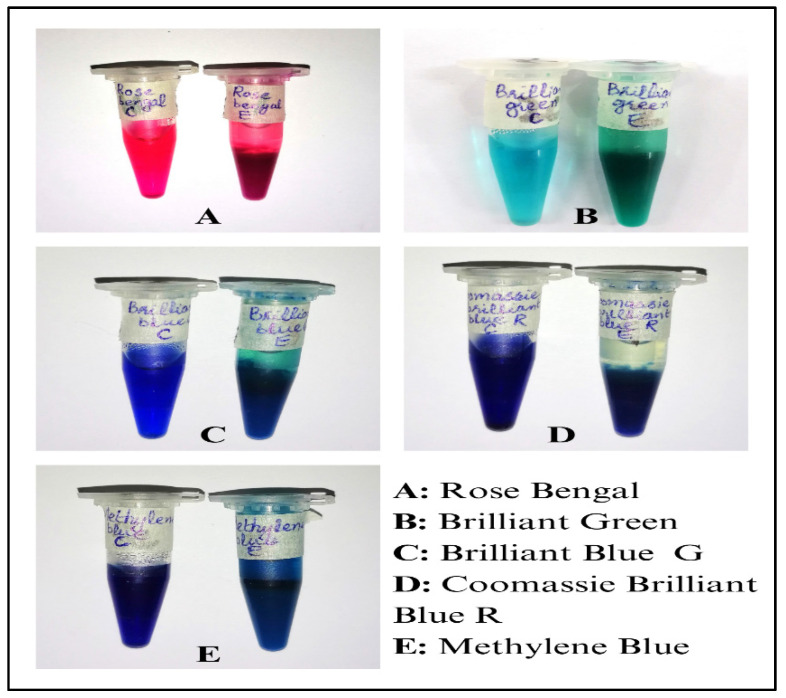
Precipitation of textile dyes carried out by recombinant laccase.

The dyes used in the present study are actively used in the textile industry and, thus, are components of effluents. The rILac actively precipitated the dyes. The dye solutions were partially precipitated within one hour and completely precipitated within two hours, while the control mixture had the same texture, and its dye was still in a soluble state. The effect of the enzyme on the dyes is shown in Figure 7.

**Figure 8:**
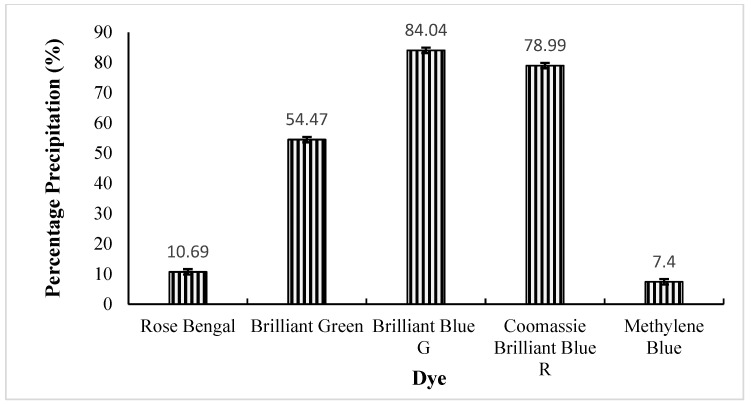
The precipitation percentage of dyes carried out by rILac.

The extent of precipitation, calculated as a percentage, is shown in Figure 8. The results show that brilliant blue G precipitated the most, followed by Coomassie brilliant blue R and then brilliant green.

#### FTIR and UV–Vis Spectrophotometric Analysis of the Dyes

The FTIR comparison of the dyes before and after treatment with rILac was carried out to probe the changes brought about by laccase.

**Figure 9:**
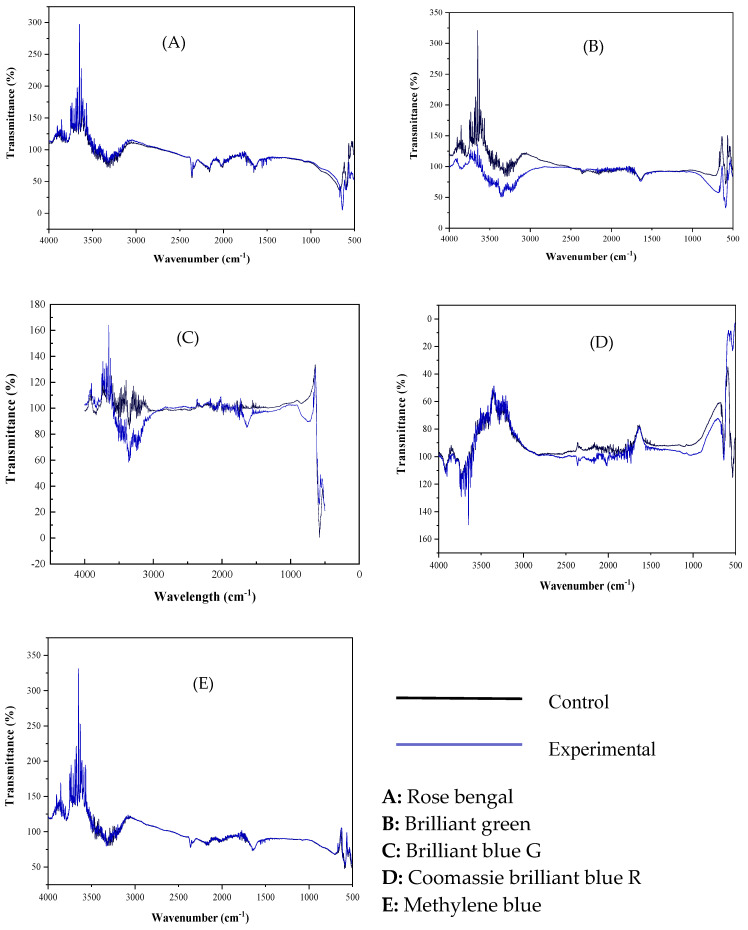
FTIR analysis of the dyes before and after treatment with rILac.

A comparison of the FTIR results of rILac-treated and untreated rose bengal dye is shown in Figure 9A. The development of new peaks in the 3500 to 4000 cm^−1^ region shows the change in the bonding of the dye. This change in the bonding is a clear indication of the action of rILac on the dye. The transmittance dropped from 1000 to 500 cm^−1^, indicating the absorption of the incident light due to the high population of bonds as the vibrational energies corresponded with the incident light.

In Figure 9B, the band at 3392 cm^−1^ is due to the N-H stretching in brilliant green. The band of C=O stretching is observed at 1641 cm^−1^. A clear drop in the transmittance after rILac treatment was observed at 4000–2526.74896 cm^−1^ and 1116.78446–500 cm^−1^ due to the dense precipitates that absorbed more light as the incident light corresponded with the vibrational energies of the bonds. The change in the transmittance in these bands confirms the degradation of brilliant green dye with rILac.

Figure 9C shows the changes brought about in the brilliant blue G. The peaks at 1641 cm^−1^ are attributed to C=O stretching, and those at 3394 cm^−1^ and 3433 cm^−1^ are due to N-H stretching. The most precipitated dye formed very dense precipitates that absorbed more light and decreased the transmittance. The increased absorption of light is caused by the high population of bonds. The comparison of the brilliant blue G dye before and after treatment showed a change in transmittance at the 4000–3741.90304 cm^−1^, 3568.3096–2229.7113 cm^−1^ and 1728.21914–500 cm^−1^ ranges, showing the change in bond nature.

A comparison of the rILac-treated and untreated Coomassie brilliant blue R dye is shown in Figure 9D. It shows a visible change in the transmittance due to the precipitation of the dye. A change in the peaks at 3884 and 3616 cm^−1^, which belongs to the O-H bond, can be observed. The change in the peak is due to the change in the nature of the bond due to the action of rILac on the dye.

Very few changes in the FTIR spectra of the methylene blue dye before and after treatment were observed (Figure 9E), as the rILac caused only slight precipitation, as mentioned earlier.

**Figure 10:**
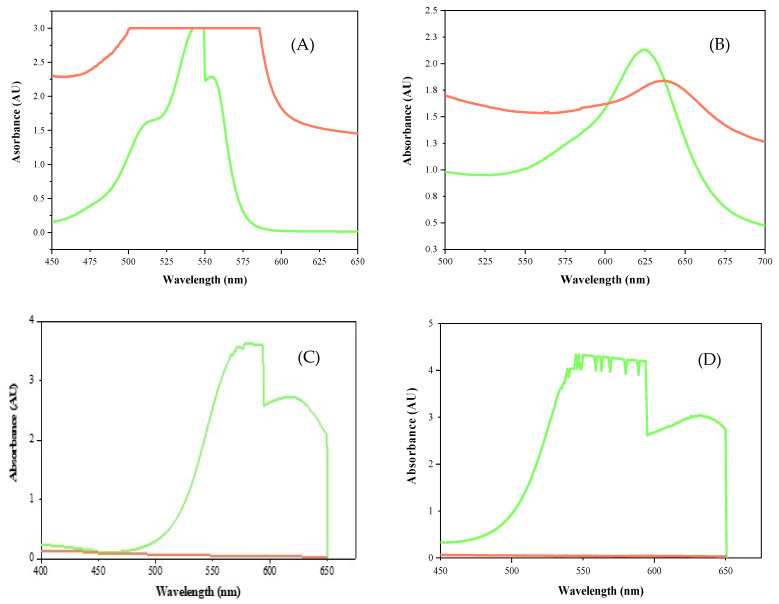

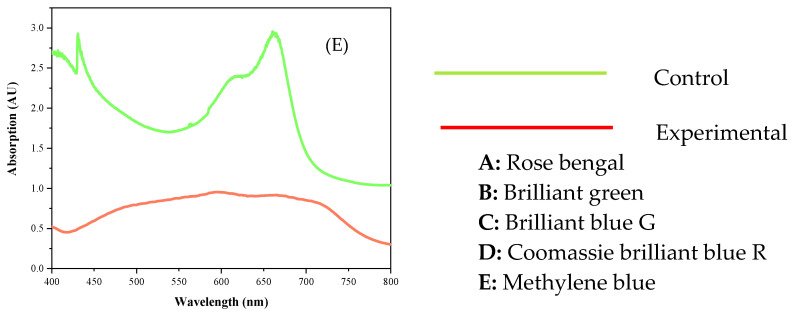
UV–Vis spectrophotometer analysis of the dyes before and after treatment with rILac.

The rILac changed the composition of the dyes and the typical behavior of the dyes for the specific wavelengths. The UV–Vis spectrophotometric results show that the absorbance peaks of all the dyes were shifted from their original position, and their shapes were also altered. All the results are a clear indication of the dye removal from the solutions. Analysis shows that rILac had changed the dye composition. Rose bengal dye shows an absorption peak at 562 nm and absorbs light in the 450–650 nm wavelength region [30]. Figure 10A shows that rILac changed the optical absorbance of the dye via precipitation, which is clear from the increase in the absorbance.

Brilliant green dye typically shows an absorbance peak at 625 nm [31]. Figure 10B shows the difference between the untreated dye and the dye treated with rILac. The shift in the absorbance peak toward 650 nm and an increase in the absorbance indicates the precipitation and a compositional change brought about by rILac.

Brilliant blue G shows an absorption peak at 587 nm. The rILac changed the composition of the dye, which is evident from the change in the absorbance peak of the treated dye shown in Figure 10C. The change in the absorbance pattern can also be seen along the absorbance peak.

Coomassie brilliant blue R shows an absorption peak at 587 nm, which vanished after treatment with rILac. The absorbance pattern also changed (Figure 10D).

Methylene blue shows an absorbance peak at 664 nm. The rILac changed the shape of the absorption peak of the dye by causing the structural changes, as shown in Figure 10E. The change in the absorbance pattern can also be observed.

## 3. Discussion

Laccase producers were screened on the basis of the oxidation of guaiacol present in the media. The bacterial colonies that oxidized guaiacol produced a brown-colored product (biphenoquinone) in the form of a brown zone around them. The larger the zone, the higher the guaiacol production of that colony [32]. The colony that produced the largest brown zone among all was determined to be the best producer. Wang et al. [33] cloned the CotA laccase gene from *Bacillus subtilis* into *E. coli* BL21 (DE3). They also found their protein to be expressed intracellularly.

The medium YPD-Cu had all the necessary components required to support the optimum growth of *E. coli*, which led to more favorable production. It contained glucose, peptone, yeast extract and copper sulfate, making it a rich source of carbon, nitrogen, essential vitamins and minerals. Glucose, being a preferred source of carbon, was an ideal option for the medium [34]. Yeast extract and peptone contain many types of minerals, vitamins and amino acids, making them a good nutritional source [35]. The amino acids in the medium also help to neutralize the pH changes brought about by the glucose and metabolic processes, as the acids produced tend to shift the pH more toward an acidic level that harms the cells [36]. The addition of divalent cations in the form of copper sulfate is helpful for microbial growth, and it being a part of the active site has a direct and positive impact on laccase production [37]. Pezella et al. [38] also found that their recombinant *Pichia pastoris* with the laccase gene showed optimal recombinant production in the YPD medium. The maximum production at 37 °C might be due to the fact that the lac promoter is maximally active at that temperature [39]. An elevated temperature causes proteolytic damage to the proteins [40]. Basheer et al. [41] also found that recombinant *E. coli* with the laccase gene from *Bacillus subtilis* exhibited a maximum yield at 37 °C. It is observed that increasing the fermentation time up to a certain limit affects the growth pattern of the microbes. However, this effect decreases after a certain point when the available nutrients are depleted, and the concentration of toxic byproducts increases [42]. The same optimization time for laccase production was mentioned by Wang et al. [43], who cloned the laccase gene from *Bacillus licheniformis* into *Pichia pastoris*. The recombinant E.coli was found to grow optimally at 7.5 pH. This might be due to the fact that *E. coli* grows maximally at 6.5–7.5 pH [44]. Niladevi et al. [45] also found 7.5 pH to be the optimum for the maximum production of laccase as the metabolic machinery works optimally at that pH. The *E. coli* consumes glucose present in the fermentation media for the metabolic processes. The metabolic machinery produces many byproducts besides the required ones. These include acetic acid, formic acid, lactic acid, ethanol, etc., which make the media more acidic. The decline in the pH below 6 ceases the *E. coli* growth and causes a decline in recombinant production [46]. These results are supported by Kalyani et al. [47], who also found glucose to be the best carbon source when they expressed the laccase gene from *Meiothermus ruber* DSM in *E. coli* BL21 (DE3). The reason for the maximum laccase production by rIBL21 codon plus in the presence of glucose lies in the fact that glucose is the preferred carbon source for all micro-organisms along *E. coli* [48]. Kontro et al. [49] reported 2% glucose to be ideal for the production of laccase when they cloned the laccase gene from *Coprinopsis cinerea* into *Pichia pastoris*. An elevated glucose concentration has a high potential to cause catabolite repression and decrease cyclic monophosphate (cAMP), which is a starvation signal. It ultimately leads to decreased recombinant enzyme production [50]. In 2022, Umar and Ahmed [51] also reported yeast extract to be the best nitrogen source among all available organic and inorganic nitrogen sources for the maximum production of laccase. Yeast extract is a natural blend of various amino acids, purines, vitamins and glutamic acid. Therefore, it efficiently helps in the production of various complex carbohydrates, metabolites and fats. This is a possible reason for the boost in rILac production. Yeast extract has been proven to be a beneficial nitrogen source for microbial growth. Besides helping microbial growth from a nutritional point of view, it provides essential amino acids for the building of structural components. Moreover, it has been found that yeast extract helps to maintain the pH of a medium due to its basic nature, as microbial activity makes the medium acidic [36].

Lactose is the natural inducer of the T7 promoter; however, it is metabolized over time, causing the cells to revert to their uninduced state. IPTG is a synthetic inducer that is not metabolized by cells, making it a good inducer enabling cells to produce recombinant protein for a long period of time [52]. Wang and Zhao [53] observed 0.1 mM IPTG to be the best concentration for optimal induction when they cloned the CotA laccase gene in *E. coli* BL21 (DE3). The suitable concentration of IPTG is dependent upon various factors like the number of lac repressor molecules, lac promoter genes and the target protein. Increasing the concentration of IPTG beyond a certain point can cause the onset of an early stationary phase [54]. Moreover, it can lead to the induction of proteases that, in turn, cause the proteolytic degradation of the recombinant proteins [55]. Premature induction prevents the positive impact on recombinant enzyme production as, at that time, the cells are in the lag phase. Very late induction is also unhelpful because, after the onset of the stationary phase, the cells start dying due to nutrient depletion and toxic waste accumulation. Thus, the induction time must be carefully determined [56]. Zhang et al. [57] cloned the laccase gene from *Bacillus subtilis* into *E. coli* BL21 DE3 and found 0.1 mM IPTG to be most appropriate for expression. Copper ions, being major components of the active site of the enzyme, must be carefully optimized as they are required in a very minute concentration. The elevated levels may generate hydroxyl radicals via Fenton and Haber–Weiss reactions, which lead to lipid, protein and DNA damage [58]. Excess copper ions can also interrupt the electron transport chain, which causes an inhibition of the substrate conversion [59]. Thus, an excessive concentration of copper ions can be directly lethal to cells.

The dyes present in wastewater negatively affect the BOD (biochemical oxygen demand), COD (chemical oxygen demand), TDS (total dissolved solids), TSS (total suspended solids) and pH, making it a significant threat to the aquatic ecosystem and human beings [60]. Whenever the laccase enzyme makes contact with phenolic compounds, it leads to the formation of aryloxy radicals. These complexes spontaneously react to form precipitates, which settle in an insoluble and dense form [61]. The same behavior was exhibited by the crude rILac in the present study. This property makes rILac more suitable for the removal of textile dyes and other harmful compounds from wastewater, as these dye precipitates can be effectively removed from effluent via filtration or decantation [62]. In the present study, brilliant blue G was precipitated the most. In the past, many researchers have worked on dye treatments, but these processes were more efficient in the presence of expensive mediators like ABTS. However, in the present research, the precipitation was carried out efficiently in the absence of mediators. For example, Sharma and Leung [63] employed laccase from *Sulfitobacter indolifex* to decolorize many dyes like alizarin, acid red 27, Congo red, bromophenol blue, Coomassie brilliant blue R, malachite green and indigo carmine in the presence and absence of mediators like ABTS with overnight incubation. They found that in the absence of a mediator, only indigo carmine was decolorized by 90%, while other dyes exhibited precipitation of less than 40%. Loncar et al. [64] cloned the laccase gene from *Bacillus amyloliquefaciens* in *E. coli*. They studied its behavior toward the dyes. They also observed that their recombinant laccase caused the precipitation of the dye over several hours.

## 4. Materials and Methods

### 4.1. Chemicals

The analytical-grade chemicals for the present study were purchased from Merck (Darmstadt, Germany), Fluka (Buchs, Switzerland) and Sigma Chemicals Co. (St. Louis, MO, USA).

### 4.2. Isolation of Laccase-Producing Bacteria from Soil

The laccase-producing bacteria were isolated from the soil sample collected from the site with soil contaminated by textile dye. The production of laccase by the native strain was confirmed by the activity against 10 mM guaiacol added to the nutrient agar medium. The strain was sent for 16S rRNA sequencing for identification.

### 4.3. Cloning of Laccase Gene from Locally Isolated Strain

After molecular identification, the laccase gene was isolated and amplified using primers (5′-CATATGAATCCTGAGCCATTAAAAAAAAGCCATCACG-3′ forward and 5′-GGATCCTTACTCCTCCTTATAGCCTATGAAGTTAAACATGCG-3′ reverse primer) with NdeI and BamHI restriction sites. The amplified gene was cloned into PCR vector 2.1 and further transformed into *E. coli* DH5α. The laccase gene was further sub-cloned into pET21a by using NdeI and BamHI restriction sites. The vector bearing the laccase gene was transformed into *E. coli* BL21 codon plus. The whole process is explained in detail [29] in a previously published research paper.

### 4.4. Expression of Recombinant Laccase

The expression of recombinant laccase was analyzed by carrying out fermentation in LB broth with 50 µg/mL ampicillin and inoculation with 2% overnight inoculum of one single positive colony. When the broth attained 0.4 O.D., it was induced with 0.1 mM IPTG and 500 mM CuSO_4_. The cells from the overnight grown culture were separated from the supernatant via centrifugation at 6000 RPM for 20 min to determine the enzyme activity in each fraction. The pellet was sonicated via resuspension in 500 mM Tris-Cl pH 7 buffer. The cells were sonicated for 30 min by setting the sonication cycle to 30 s burst and 30 s off. The cell lysate was obtained after centrifugation for enzyme activity analysis. The enzyme activity was determined in both the supernatant and cell lysate by following the protocol of Peter et al. [65]. The reaction mixture contained 50 mM guaiacol, 100 mM sodium acetate buffer (pH 5.5) and the enzyme. The change in O.D. was observed at 530 nm. The enzyme activity of laccase is defined as the amount of enzyme required to catalyze one micromole of substrate per minute [66]. The formula for the determination of enzyme activity is provided below.

$$Enzyme\;activity\left(\frac{u}{mL}\right)=\frac{∆A_{530}nm/\min\times V_{t}\times dilution\;factor}{\varepsilon \times V_{s}}$$

where V_t_ = final volume of reaction mixture (mL); V_s_ = sample volume (mL); ε = extinction co-efficient of guaiacol (62.5).

### 4.5. Optimization of Laccase Production from Recombinant E. coli

Various fermentation parameters were extensively optimized to increase the rILac production. These parameters included medium (**LMI:** glucose 40 g/L, glycerol 7 g/L, L-histidine 0.50 g/L, NaNO_3_ 1.80 g/L, CuSO_4_ 0.10 g/L, NaCl 1.80 g/L, KCl 0.50 g/L, CaCl_2_·H_2_O 0.50 g/L, FeSO_4_·7H_2_O 0.05 g/L, KH_2_PO_4_ 1.00, MgSO_4_·7H_2_O 0.50 g/L; **LMII:** glucose 10.0 g/L, peptone 3.0 g/L, KH_2_PO_4_ 0.6 g/L, K_2_HPO_4_ 0.4 g/L, ZnSO_4_ 0.001 g/L, FeSO_4_ 0.0005 g/L, MnSO_4_ 0.05 g/L, MgSO_4_ 0.5 g/L, CuSO4 0.01 g/L; **LMIII:** peptone 3 g/L, glucose 10 g/L, KH_2_PO_4_ 0.6 g/L, K_2_HPO_4_ 0.4 g/L, ZnSO_4_ 0.001 g/L, FeSO_4_ 0.0005 g/L, MnSO_4_ 0.05 g/L, MgSO_4_ 0.5 g/L; **LMIV:** potassium hydrogen phthalate 0.04 g/L, NaNO_3_ 2.6 g/L, K_2_HPO_4_ 0.4 g/L, KH_2_PO_4_ 0.6 g/L, MgSO_4_·7H_2_O 0.5 g/L, NaCl 0.5 g/L; **LMV:** glucose 10.0 g/L, Asparagine 0.50 g/L, yeast extract 0.50 g/L, K_2_HPO_4_ 0.50, MgSO_4_·7H_2_O 1.00, FeSO_4_·7H_2_O 0.01; **LMVI (Terrific broth):** bacto-tryptone 12 g/L, bacto-yeast 24 g/L, glycerol 4 mL, (0.17 M KH_2_PO_4_ + 0.72 M K_2_HPO_4_ = 100 mL); **LMVII (YPD-Cu medium):** glucose 20 g/L, peptone 5 g/L, yeast extract 2 g/L, CuSO_4_ 100 mg/L; **LMVIII (LB broth):** yeast extract 5.0 g/L (*w*/*v*), tryptone 10.0 g/L (*w*/*v*), NaCl 10.0 g/L (*w*/*v*); **LMIX (Super broth):** tryptone-bacto 32 g/L, yeast extract 20 g/L, NaCl 5 g/L; **LMX (TYGPN medium):** tryptone 20 g/L, yeast extract 10 g/L, glycerol 80% 10 mL/L, Na_2_HPO_4_ 5 g/L, KNO_3_ 10 g/L), temperature (31, 33, 35, 37, 39 and 41 °C), fermentation time (12–72 h with a 6 h gap), pH (5.0, 5.5, 6.0, 6.5, 7.0, 7.5, 8.0, 8.5 and 9.0), carbon source (sucrose, fructose, glucose, maltose, mannitol, starch, lactose, dextrose, glycerol, galactose and saccharose) and concentration. The inorganic (ammonium sulfate, sodium citrate, calcium nitrate, ammonium chloride, ammonium nitrate and sodium nitrate) and organic (peptone, yeast extract, tryptone, urea, glycine, tryptophan and skimmed milk) nitrogen sources and their optimum concentrations were also determined. The inoculum size (1–5 mL), inducer type, inducer concentration, induction time (0.4, 0.5, 0.6, 0.7 and 0.8 O.D.) and copper ion concentration were also optimized.

### 4.6. Dye Removal

The activity of the produced rILac was assessed to determine its efficiency against solutions of various dyes. The reaction mixture was prepared with three components, namely, sodium acetate buffer, rILac and dye. The various dyes employed for this purpose included rose bengal, brilliant green, brilliant blue G, Coomassie brilliant blue R and methylene blue, which are actively used in the textile industry. The final concentration of these dyes was set to 100 mg/L by dissolving them in distilled water, and the final concentration of the rILac in the reaction mixture was 1 U/mL. On the other hand, the control mixture contained only sodium acetate buffer (pH 5.5) and dye. The effect of the recombinant enzyme on the dye was studied under standard assay conditions [67]. The change in O.D. for the dyes rose bengal, brilliant green, brilliant blue G, Coomassie brilliant blue R and methylene blue was studied at 562, 625, 587, 587 and 664 nm, respectively. The precipitation percentage of dye was calculated using the following formula [68].

$$\%\;Precipitation=\frac{O.D.Initial-O.D.Final}{O.D.Initial}\times 100$$

O.D. initial = O.D. before precipitation

O.D. final = O.D. after precipitation

#### FTIR and UV–Vis Spectrophotometric Analysis of the Dyes

The precipitated dyes were analyzed with FTIR spectroscopy for changes in the chemical structure of the dyes before and after treatment with rILac.

The extent of precipitation was studied in detail via UV–Vis spectrophotometric analysis of the dyes before and after treatment with the dyes. The rose bengal dye exhibits an absorbance peak at 562 nm; therefore, the changes in the absorbance pattern before and after treatment were studied in the 450–650 nm region [30]. Brilliant green dye exhibits an absorbance peak at 625 nm. Therefore, the absorbance pattern was studied at 400–700 nm [69]. Brilliant blue G, Coomassie brilliant blue R and methylene blue show absorbance peaks at 587 nm [70,71] and 664 nm [63].

## 5. Conclusions

The laccase gene cloned from *B. megaterium* resulted in a successful clone, the production of which was optimized. The optimized recombinant enzyme very efficiently removed textile dyes from the water via precipitation. This useful application of rILac can be further studied and optimized in the future, given the global need for sustainable enzymatic treatment of wastewater. In this way, environmental pollution, which is a main concern due to its potent health hazards, can be overcome.

## Data Availability

Data are contained within the article.
